# Sleep disturbances, sleep quality, and cardiovascular risk factors in women with polycystic ovary syndrome: Systematic review and meta-analysis

**DOI:** 10.3389/fendo.2022.971604

**Published:** 2022-09-13

**Authors:** Jiayu Zhang, Jiawen Ye, Xinge Tao, Wenjing Lu, Xueqin Chen, Changqin Liu

**Affiliations:** ^1^ School of Nursing, Fujian University of Traditional Chinese Medicine, Fuzhou, China; ^2^ The Third Clinical Medical College, Fujian Medical University, Fuzhou, China; ^3^ School of Medicine, Xiamen University, Xiamen, China; ^4^ The First Affiliated Hospital of Xiamen University, School of Medicine, Xiamen University, Xiamen, China; ^5^ Department of Endocrinology and Diabetes, The First Affiliated Hospital of Xiamen University, School of Medicine, Xiamen University, Xiamen, China; ^6^ Fujian Province Key Laboratory of Diabetes Translational Medicine, The First Affiliated Hospital of Xiamen University, School of Medicine, Xiamen University, Xiamen, China; ^7^ Xiamen Diabetes Prevention and Control Center, the First Affiliated Hospital of Xiamen University, School of medicine, Xiamen University, Xiamen, China

**Keywords:** Polycystic ovary syndrome, sleep disturbance, cardiovascular risk factors, systematic review, meta-analysis

## Abstract

**Objectives:**

(1) To establish the prevalence of sleep disorders in women with PCOS. (2) To establish the association between sleep disturbance and cardiovascular risk factors in women with PCOS.

**Methods:**

The electronic databases PubMed and EMBASE were searched for observational studies of individuals with PCOS published in English from inception to 21 October 2021. The dichotomous outcome measure was presented as odds ratio (OR) and 95% confidence interval (CI). The mean difference (MD) in continuous variables was expressed for each study.

**Results:**

A total of 18 articles were included in this meta-analysis, with a total of 16,152 participants from nine different countries. Women with PCOS had a high prevalence of sleep disturbance (OR = 6.22; 95% CI: 2.77, 13.97; *p* < 0.001), higher PSQI scores (MD = 2.10; 95% CI: 0.29, 3.90; *p* = 0.02), and shorter duration of sleep (MD = −15.65 min; 95% CI: −27.18, −4.13; *p* = 0.008). We found that body mass index (BMI), systolic blood pressure (SBP), diastolic blood pressure (DBP), low-density lipoprotein cholesterol (LDL-c), fasting glucose, 2-h glucose, and waist circumference (WC) levels were significantly higher and high-density lipoprotein cholesterol (HDL-c) was significantly lower in PCOS with sleep disturbance than in PCOS without sleep disturbance.

**Conclusions:**

The current study shows a high prevalence of sleep disturbance in women with PCOS and provides evidence of an association between cardiovascular risk factors and sleep disturbance among this population. Increased attention should be paid to sleep management in clinical guidelines for PCOS.

**Systematic Review Registration:** https://www.crd.york.ac.uk/PROSPERO/, identifier CRD42022298040.

## 1 Introduction

Polycystic ovary syndrome (PCOS) is a prevalent disorder affecting 8%–13% of reproductive-age women (1). Modifying lifestyle behaviors such as weight reduction, physical activity, and dietary interventions have become central to managing this condition (2). Sleep is an important lifestyle factor essential to a woman’s health and wellbeing (3). However, women with PCOS appear to have an increased frequency of sleep problems (4–6). Sleep disturbances, including difficulty in falling asleep, early morning awakenings, and/or altered sleep duration, have profound negative unintended consequences on the cardiovascular system with increased prevalence of hypertension, coronary heart disease, and stroke (7, 8). Obstructive sleep apnea (OSA) and sleep-disordered breathing (SDB) were characterized by fragmentary sleep, and both have previously been linked with PCOS (9, 10). Moreover, sleep patterns may be influenced by shiftwork or jet lag, both of which are associated with increased cardiovascular risk (11). Consequently, there is increased recognition that sleep assessment is not mentioned in clinical guidelines for PCOS.

Although studies on PCOS and sleep have been extensively carried out in the last decade, the theoretical perspective that sleep is directly affected by the unique biology of PCOS is relatively new. Hyperinsulinemia and hyperandrogenism play an essential foundational role in the pathogenesis of PCOS. Both of them have been shown to alter the expression of circadian clock genes, especially brain and muscle ARNT-like protein 1 (BMAL1), period (PER) 1, and PER2 (12, 13), which are closely intertwined with sleep and wake cycles (14). Dysregulation of clock gene expression also affects the temporal molecular regulation of metabolism, thereby aggravating metabolic dysfunction (15). Recent research has found that poor sleep behaviors are associated with the development of metabolic disease in adolescents with PCOS (4). Intermittent hypoxia caused by fragmented sleep can lead to tissue hypoxia and trigger a series of reactions such as oxidative stress, mitochondrial dysfunction, and inflammation (16). Thus, poor sleep behaviors over a longer period can negatively affect metabolic function. This will increase the risk of metabolic syndrome in PCOS.

Previous cross-sectional studies have investigated the relationship between PCOS and the prevalence of sleep disturbance (17–20). In a cross-sectional study of 87 women with PCOS, the prevalence of poor sleep was high at 31% (19). Bennett and colleagues reported that women with PCOS had more severe adverse sleep symptoms compared with those without PCOS (20). Chatterjee et al. reported that 66% of 50 women with PCOS had sleep-disordered breathing (21). However, findings from a prospective cross-sectional study indicated that no significant associations were observed between sleep duration and the level of androgenic hormones (17). Thus, the outcomes of previous clinical studies were inconclusive and inconsistent. Also, most of the reviews about PCOS have mainly focused on psychological distress such as anxiety and depression, but whether there is a relationship between cardiovascular risk factors and sleep disturbance in women with PCOS is unknown.

Therefore, a comprehensive analysis of the relationship between cardiovascular risk factors and sleep disturbance in PCOS is required to provide researchers with the most up-to-date information. This study aimed to (1) establish the prevalence of sleep disorders in women with PCOS, and (2) establish the association between sleep disturbance and cardiovascular risk factors in women with PCOS.

## 2 Methods

### 2.1 Literature search and study selection

This meta-analysis was conducted according to the PRISMA guideline (22) (registration number: CRD42022298040). We searched PubMed and Embase for studies published by October 2021 to identify relevant articles. We used the following search terms: (“polycystic ovary syndrome” OR “syndrome, polycystic ovary*” OR “Stein–Leventhal Syndrome” OR “PCOS”) AND (“Sleep Wake Disorders” OR “Sleep Initiation and Maintenance Disorders” OR “Disorders of Initiating and Maintaining Sleep” OR “Disorder”, “Sleep Wake” OR “Sleep*”).

Articles were included if the sleep length, prevalence of sleep disturbance, or Pittsburgh sleep quality index (PSQI) scores were reported in patients with PCOS. We have also included articles if two groups of women with PCOS were based on the presence/absence of sleep disturbance. Observational studies such as case–control, longitudinal cohort, and cross-sectional studies were included in the analysis. Articles published as conference abstracts, commentary, reviews, and case reports were excluded. Only articles written in English were included.

### 2.2 Data extraction

Two investigators (JZ and JY) independently reviewed the search results, and selected articles to extract study data. Any disagreements or problems were resolved by the third researcher (CL). Standardized Excel sheet forms were designed to capture all relevant information required for analyses, including first author, year of publication, study design, country of publication, type of sleep problem, sample size, mean body mass index (BMI), mean age, diagnostic criteria for PCOS, diagnostic criteria for sleep problems, and quality assessment.

### 2.3 Study outcomes

The primary outcomes were the prevalence of sleep disturbance, PSQI scores, and sleep length in women with PCOS compared with women without PCOS. The secondary outcomes were to explore how cardiovascular risk factors influence the prevalence of sleep disturbance in women with PCOS. These were explored through analysis for BMI, systolic blood pressure (SBP), diastolic blood pressure (DBP), high-density lipoprotein cholesterol (HDL-c), low-density lipoprotein cholesterol (LDL-c), fasting glucose, 2-h glucose, and waist circumference (WC).

### 2.4 Quality assessment

Two investigators (JZ and JY) evaluated the methodological quality using the Newcastle–Ottawa Scale (NOS) for case–control and cohort studies (available at http://www.ohri.ca/programs/clinical_epidemiology/oxford.asp). Studies with scores of 7–9, 5–6, and <4 were considered as high, moderate, and low quality, respectively. The Agency for Healthcare Research and Quality (AHRQ) scale was applied for cross-sectional studies (available at https://www.ncbi.nlm.nih.gov/books/NBK35156/). The tool of AHRQ for each study ranged from 0 to 11. Studies with a score of ≥8, 4–7, and ≤3 were considered as high, moderate, and low quality, respectively.

### 2.5 Statistical analysis

We used Review Manager 5.4 to conduct all analyses (23). The dichotomous outcome measure was presented as odds ratio (OR) and 95% confidence interval (CI). The mean difference (MD) in continuous variables was expressed in women with PCOS compared to women without PCOS for each study. *I* ^2^ > 50% was considered high heterogeneity (the random-effects model was used), and values lower than 50% indicated low heterogeneity (the fixed-effects model was used) (24). We then divided these studies into subgroups according to different sleep issues. We assessed the effect of these variables in explaining heterogeneity in the prevalence of sleep disturbance. Variables that were significant at ≤ 0.05 were considered significant confounders.

## 3 Results

### 3.1 Literature search

**Figure 1:**
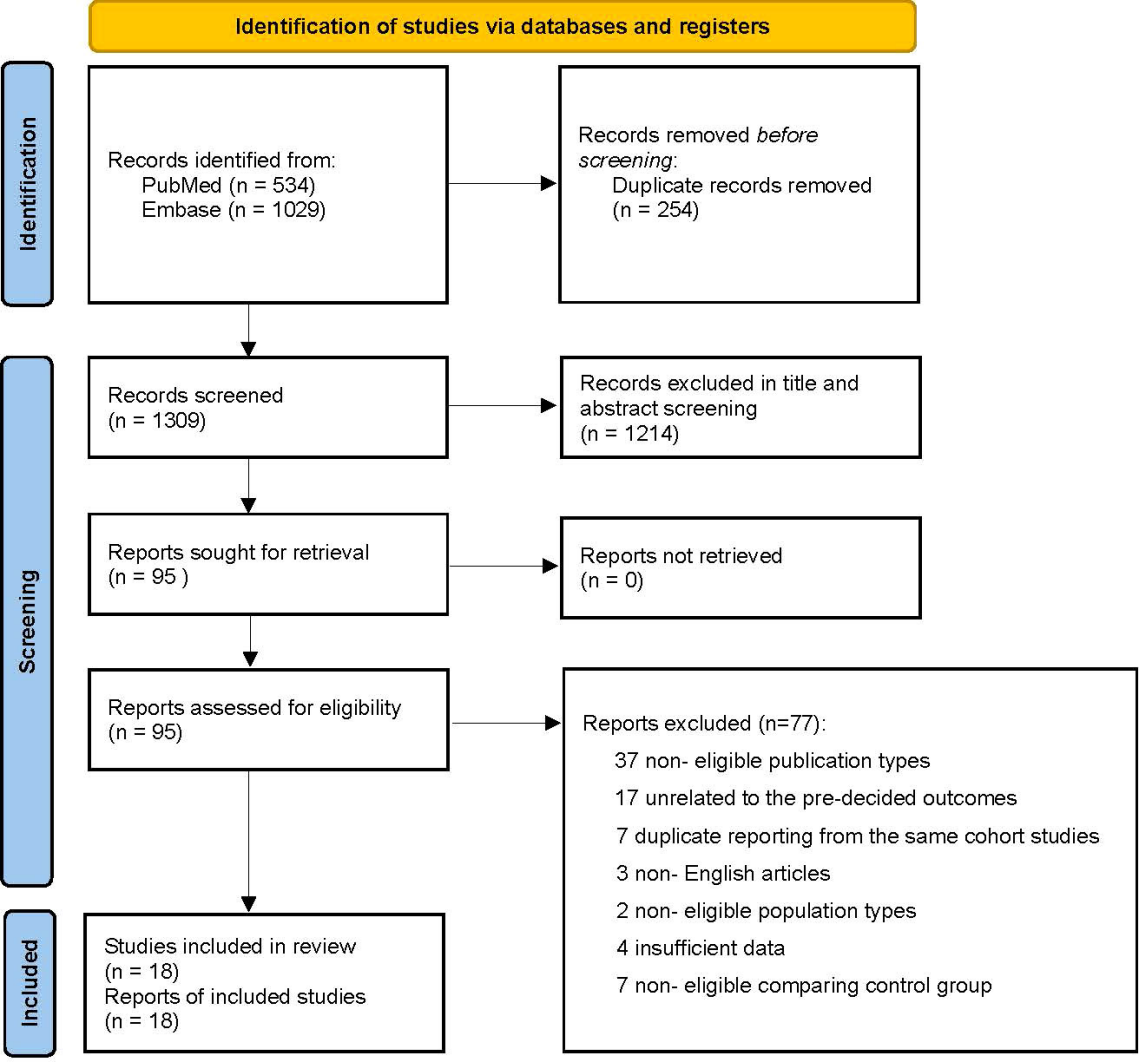
PRISMA flow diagram showing study selection.

The results of the literature selected and screened process are summarized in **Figure 1**. The literature searched totaled 1,563 records. In all, 1,468 records were excluded because they did not meet the inclusion criteria. A total of 95 full-text articles were assessed for eligibility. Seventy-seven were excluded because they were non-observational studies, were unrelated to the pre-decided outcomes, had the same outcome/duplicate report from the same cohort studies, were non-English language articles, had non-eligible population types, had a non-eligible control group in comparison, and had insufficient data. Finally, 18 full-text articles were included in the meta-analysis.

### 3.2 Characteristics of included studies

In total, 16,152 participants were included in 18 studies (18, 19, 21, 25–39). These 18 studies were published from 2001 to 2021, which included 2 cohort studies and 16 cross-sectional studies, and were eligible for the meta-analysis. Among these included studies, nine studies have reported the prevalence of sleep disturbance, seven studies have reported the sleep length, four studies have evaluated the PSQI scores, and six studies have investigated the relationship between separate sleep disturbance and cardiovascular risk factors in PCOS women. About the country of publication, seven studies were from the United States of America (USA), two studies were from Brazil, two studies were from Australia, two studies were from India, and the remaining five studies were from China, Iran, the United Kingdom (UK), Turkey, and Germany. The main characteristics of eligible studies are summarized in **Table 1**.

**Table 1:** Characteristics of included studies.

First Author	Year	Study design	Country	Type of sleep problem	Sample size(case/control)	Mean BMI(case/control)	Mean Age(case/control)	PCOSdefinition	Sleep disorder definition	Quality Assessment
Azizi	2020	cross-sectional	Iran	/	201/199	22.73(9.62)/23.95(4.96)	27.86(5.84)/28.06(6.51)	Rotterdam criteria	/	7
Chatterjee	2014	cross-sectional	India	SDB	50(33/17)	29.8(3.4)/24.36(2.29)	/	Rotterdam criteria	PSG	6
De	2011	cross-sectional	Germany	OSA	31/19	32.7(6.2)/32.4(4.0)	15.0(1.0)/15.2(1.1)	NIH	PSG	5
Fogel	2001	cross-sectional	America	OSA	18/18	36.9(1.3)/36.9(1.4)	31.1(1.3)/32.3(1.3)	Chronic oligomenorrhea along with elevated serum androgen levels	PSG	7
Hachul	2019	cross-sectional	Brazil	OSA	30/14	34.3(1.1)/22.4(1.6)	29.7(1.2)/27.9(1.7)	Rotterdam criteria	PSG,Questionnaire	7
Kahal	2020	cross-sectional	UK	OSA	39(15/24)	37.3(7.3)/32.2(7.8)	33(26-43)/29.5(27-33)	Rotterdam criteria	Questionnaire	7
Karasu	2021	cross-sectional	Turkey	/	111/108	26.47(5.10)/26.5(5.0)	25.13(5.82)/26.4(9.4)	Rotterdam criteria	Questionnaire	5
Mo	2019	Cohort	Australia	Sleep disturbances	484/6094	28.7(7.4)/25.6(5.7)	33.5(1.5)/33.7(1.4)	Self-report	Self-report	5
Moran	2015	cross-sectional	Australia	Poor sleep	87/637	30.1(25.1-38.6)/25.4(22.4-29.9)	30.2(29.9-30.8)/30.2(29.9-30.9)	Rotterdam criteria	Questionnaire	9
Nandalike	2012	cross-sectional	America	OSA	28/28	44.8(8.8)/40.2(4.7)	16.8(1.9)/17.1(1.8)	Rotterdam criteria	PSG	7
Simon	2019	cross-sectional	America	/	59/33	/	15.7(1.8)/15.8(1.4)	NIH	Questionnaire	6
Sirmans	2014	cross-sectional	America	OSA	1689/5067	/	25.24/25.23	Read codes	/	8
Su	2017	cohort	China	/	129/156	/	29.03(3.26)/31.72(3.86)	Rotterdam criteria	Questionnaire	7
Suri	2016	cross-sectional	India	SDB	50/100	28.0(4.01)/25.3(2.93)	27.9(6.44)/28.3(6.05)	Rotterdam criteria	PSG	6
Tasali	2008	cross-sectional	America	OSA	52/21	39.2(1.0)/36.0(1.5)	29.7(0.7)/30.7(1.1)	NIH	PSG	8
Tock	2014	cross-sectional	Brazil	OSA	12/26	37.8(4.8)/30.67(7.7)	28.3(5.0)/28.4(7.5)	Rotterdam criteria	PSG	6
Vgontzas	2001	cross-sectional	America	SDB	53/452	38.7(1.1)/26.4(0.3)	30.4(0.9)/32.1(0.3)	Presence of chronic anovulation association with elevated circulating androgen levels	PSG	6
Vgontzas	2006	cross-sectional	America	/	42/15	38.7(1.4)/23.3(0.5)	29.6(0.9)/31.9(1.5)	Presence of chronic anovulation with elevated circulating androgen levels	Evaluated for one night in the sleep laboratory	6

PSG, Polysomnography; NIH, National Institutcs of Health; SDB, sleep disordered-breathing; OSA, Obstructive Sleep Apnea.

### 3.3 Study quality

The quality assessment scores for the two cohort studies ranged from 5 to 7, with an average score of 6.00 points. There was a total of 16 cross-sectional studies, and the scores from the assessment of study quality ranged from 5 to 9, with a mean score of 6.63 points.

### 3.4 Prevalence of sleep disturbances

**Figure 2:**
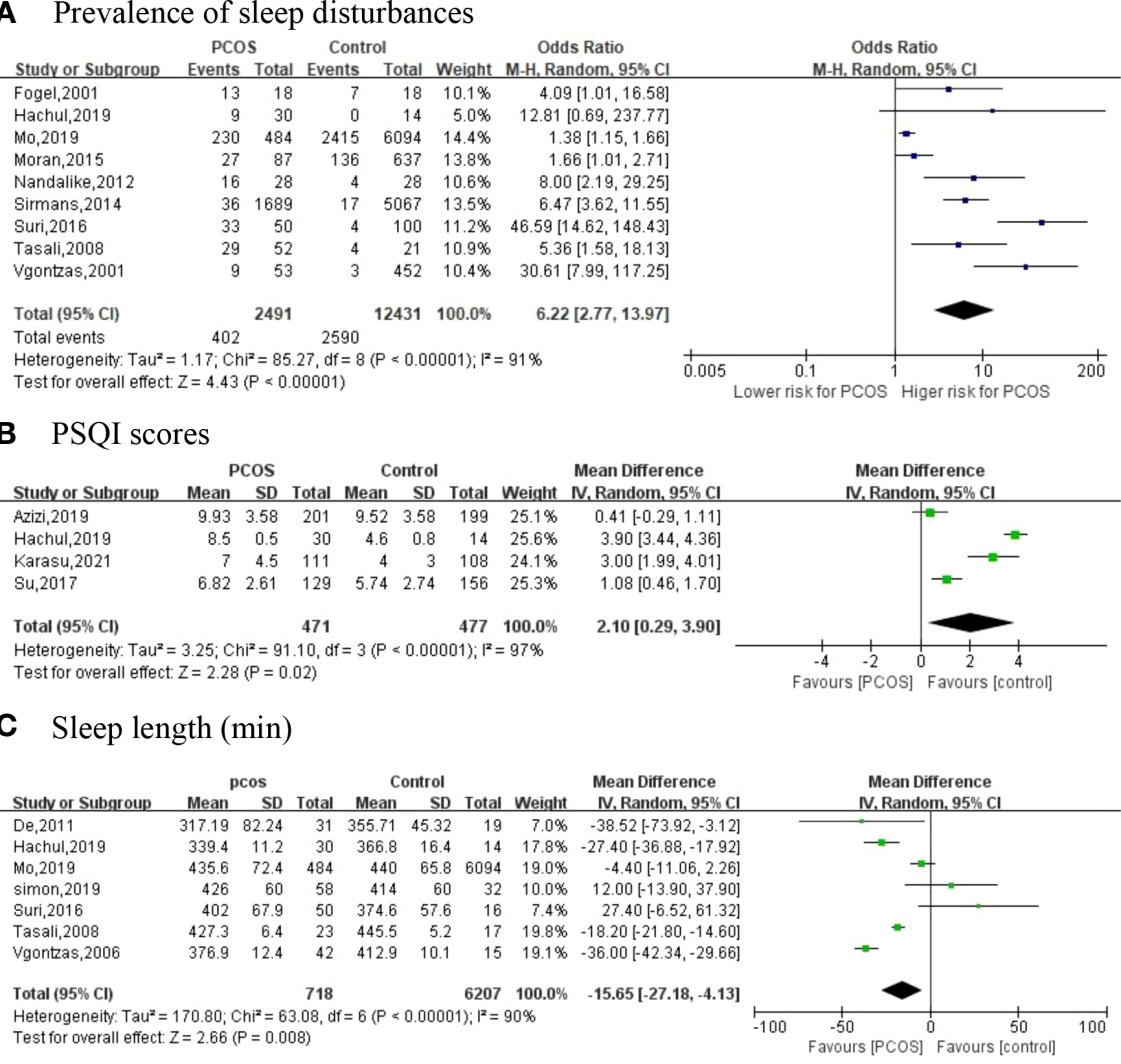
The primary outcomes [**(A)** Prevalence of sleep disturbances; **(B)** PSQI scores; **(C)** Sleep length (min)].

**Figure 3:**
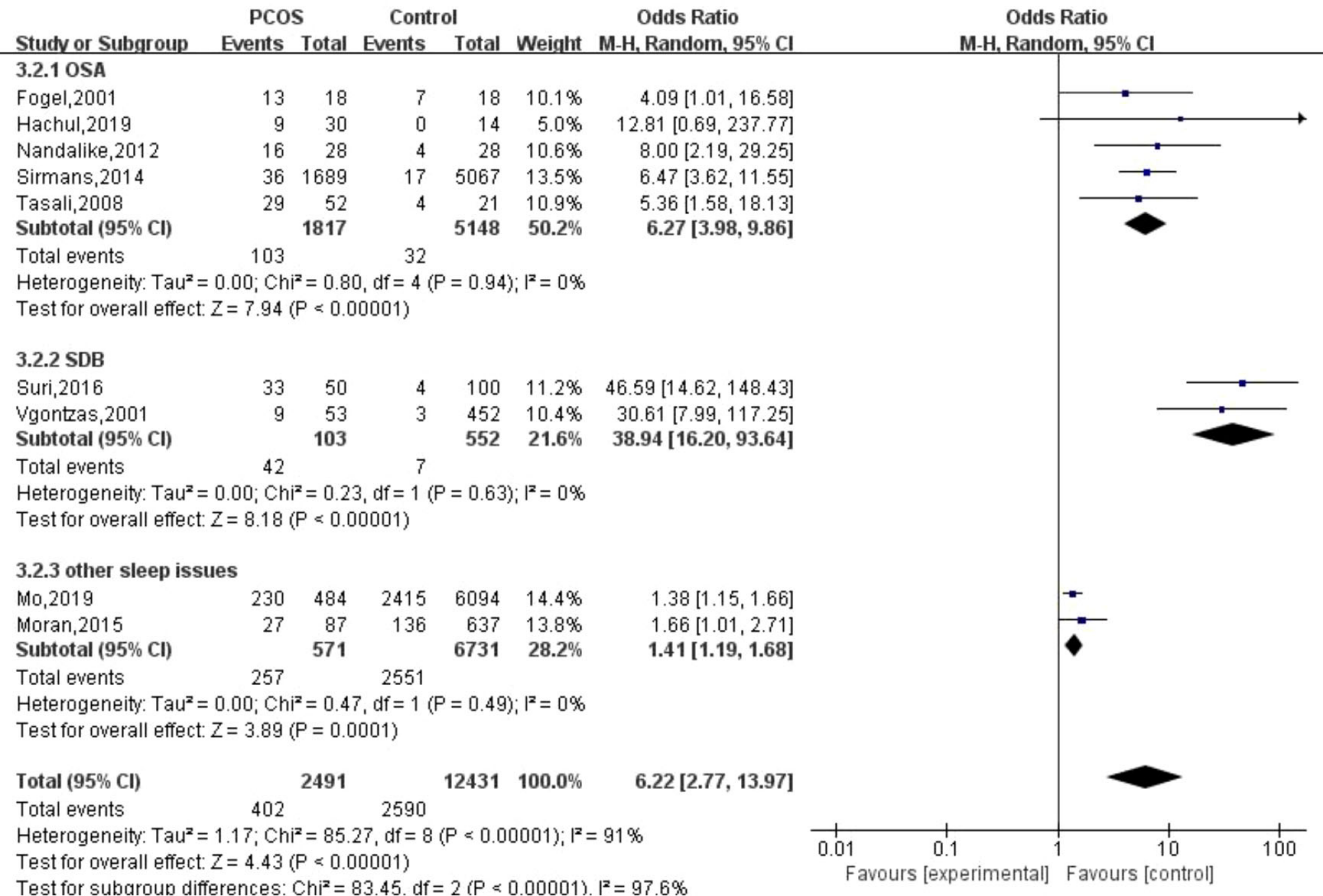
Subgroup analysis of sleep disturbances.

We included nine studies (19, 27, 28, 30, 31, 33, 35, 36, 38) that explored sleep disturbance events in women with PCOS; 95% CI from individual studies with a pooled estimate is shown in **Figure 2A**. The results showed that the prevalence of sleep disturbance in PCOS was 16.14% (402/2,491). The pooled OR in PCOS vs. control was 6.22 (95% CI: 2.77, 13.97; *p* < 0.001), which indicated that PCOS participants had a 6.22-fold risk of sleep disturbance compared to non-PCOS participants. We divided nine studies into three categories according to different types of sleep problems. Subgroup analysis was manipulated based on three categories (**Figure 3**). All studies in SDB (OR = 38.94; 95% CI: 16.20, 93.64; *p* < 0.001), OSA (OR = 6.27; 95% CI: 3.98, 9.86; *p* < 0.001), and other sleep issues (OR = 1.41; 95% CI: 1.19, 1.68; *p* < 0.001) showed a greater prevalence of sleep disturbance in PCOS.

### 3.5 Sleep quality (evaluated by PSQI scores)

Of the 18 included studies, 4 have investigated PSQI scores as an outcome between two groups (25, 28, 29) (**Figure 2B**). Four articles with high heterogeneity (*I* ^2^ = 97%; *p* < 0.001) and the random-effects model were used. PSQI scores (MD = 2.10; 95% CI: 0.29, 3.90; *p* = 0.02) are higher in women with PCOS compared to controls.

### 3.6 Sleep length

A total of seven articles (26, 28, 30, 32, 35, 36, 39) reported the sleep length (**Figure 2C**). Meta-analysis suggested that, in comparison with controls, PCOS women had a shorter duration of sleep with an MD of −15.65 min (95% CI: −27.18, -4.13; *p* = 0.008, *I* ^2^ = 90%).

### 3.7 Influences of sleep disturbances in women with PCOS on cardiovascular risk factors

**Figure 4:**
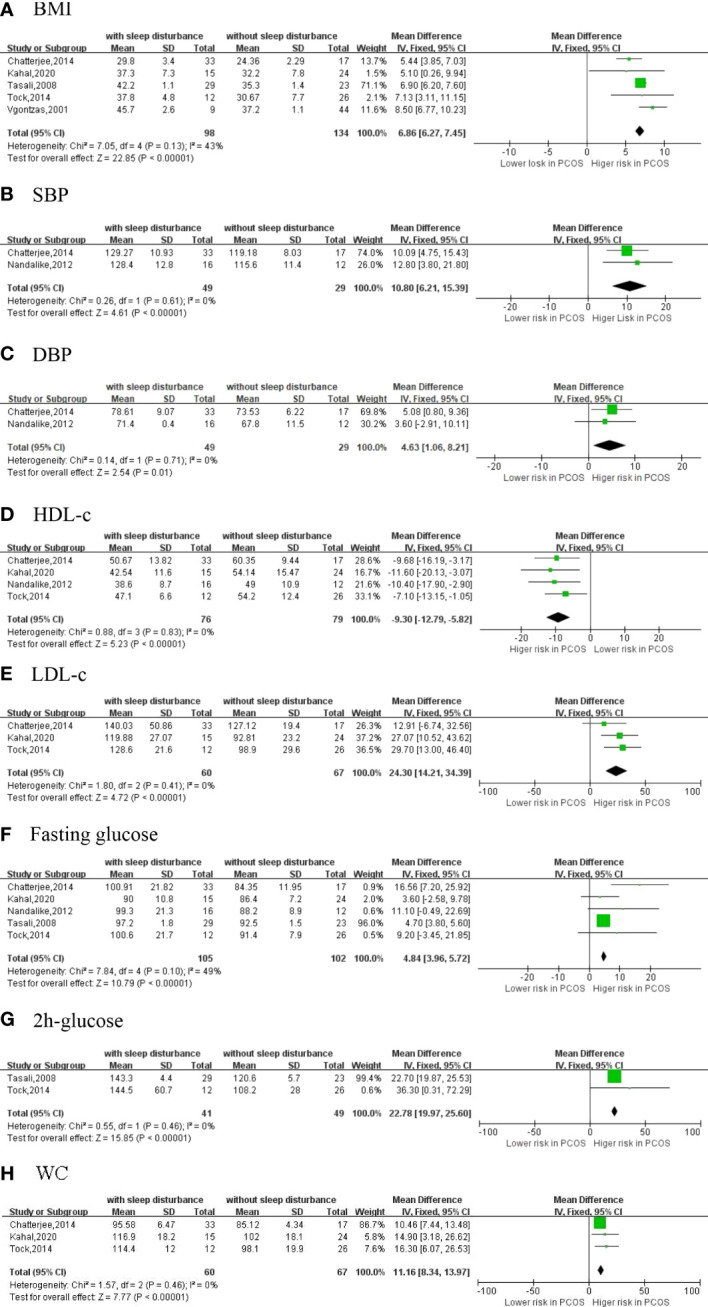
The secondary outcomes [**(A)** BMI; **(B)** SBP; **(C)** DBP; **(D)** HDL-c; **(E)** LDL-c; **(F)** Fasting glucose; **(G)** 2h-glucose; **(H)** WC].

We identified six studies (18, 21, 31, 36–38) that reported aspects of sleep disturbance and cardiovascular risk factors in PCOS women (**Figure 4**).

#### 3.7.1 Body mass index

BMI as a representative factor of general obesity was reported in five studies with 232 PCOS women. In the pooled analysis, BMI was significantly higher in PCOS women with sleep disturbance than in PCOS without sleep disturbance (MD = 6.86 kg/m^2^; 95% CI: 6.27, 7.45; *p* < 0.001, *I* ^2^ = 43%).

#### 3.7.2 Blood pressure

Blood pressure was reported in two studies with 78 PCOS women. In the pooled analysis, SBP (MD = 10.80 mmHg; 95% CI: 6.21, 15.39; *p* < 0.001, *I* ^2^ = 0%) and DBP (MD = 4.63 mmHg; 95% CI: 1.06, 8.21; *p* = 0.01, *I* ^2^ = 0%) were significantly higher in PCOS women with sleep disturbance than in PCOS controls with normal sleep.

#### 3.7.3 Blood lipid profile

LDL-c was reported in three studies with 127 PCOS women. In the pooled analysis, LDL-c (MD = 24.30 mg/dl; 95% CI: 14.21, 34.39; *p* < 0.001, *I* ^2^ = 0%) was significantly higher in PCOS women with sleep disturbance than in PCOS controls with normal sleep. HDL-c was reported in four studies with 155 PCOS women. HDL-c was significantly lower in PCOS women with sleep disturbance than in controls (MD = −9.30 mg/dl, 95% CI: −12.79, −5.82; *p* < 0.001, *I* ^2^ = 0%).

#### 3.7.4 Blood glucose

Fasting glucose was reported in five studies with 207 PCOS women. In the pooled analysis, fasting glucose (MD = 4.84 mg/dl; 95% CI: 3.96, 5.72; *p* < 0.001, *I* ^2^ = 49%) was significantly higher in PCOS women with sleep disturbance than in PCOS controls with normal sleep. Two-hour glucose was reported in two studies and was significantly higher in PCOS women with sleep disturbance than in controls (MD = 22.78 mmol/L; 95% CI: 19.97, 25.60; *p* < 0.001, *I* ^2^ = 0%).

#### 3.7.5 Waist circumference

WC as a representative factor of central obesity was reported in three studies with 127 PCOS women. In the pooled analysis, WC was significantly higher in PCOS women with sleep disturbance than in those without (MD = 11.16 cm; 95% CI: 8.34, 13.97; *p* < 0.001, *I* ^2^ = 0%).

## 4 Discussion

This systematic review and meta-analysis is the first study to comprehensively explore sleep duration and quality in women with PCOS. The review included 2 cohort studies and 16 cross-sectional studies of a total of 16,152 participants showing a high proportion of sleep problems, including short sleep duration, poor sleep quality, and high prevalence of sleep disturbance in women with PCOS. Based on the existing six prospective studies, the meta-analysis revealed the association between sleep disturbance and cardiovascular risk factors in PCOS women. We also found that BMI, SBP, DBP, LDL-c, fasting glucose, 2-h glucose, and WC levels were significantly higher and HDL-c was significantly lower in PCOS with sleep disturbance than in PCOS without sleep disturbance.

According to the current study, the majority of PCOS women experience sleep disturbance (16.14%, 402/2,491) (**Figure 2A**). Additionally, we found that PCOS participants had a 6.22-fold risk (OR) of sleep disturbance compared with non-PCOS participants. The ratio was higher than that in a previous study (40), which reported that women with PCOS were 3.83 times (OR) more likely to have developed OSA than controls. A higher prevalence of anxiety in PCOS has been recognized as a consequence of sleep disturbance, as it can reduce women’s quality of life and lead to a depressed mood. Furthermore, depression caused by the pain of infertility and reproductive issues in PCOS women is also responsible for sleep disturbance (41). The PSQI is a cornerstone tool for the assessment of sleep quality. Higher scores indicate worse sleep quality (42). We discovered that sleep quality was lower in PCOS women, as the average total PSQI scores increased significantly by 2.10 units (*p* = 0.02), which is consistent with previous studies. In a study by Bennet et al. (20), an Australian longitudinal cohort of 6,057 patients reported greater adverse sleep quality in women with PCOS. Among them, in the PCOS women’s group, 37.6% experienced restless sleep, 43.9% suffered from difficulty sleeping, 56.9% felt severe tiredness, and 47.7% reported difficulty in falling asleep. A trial published in 2021 (43) concluded a strong association between insulin resistance and OSA in women with PCOS. Poor sleep quality results in insulin resistance and obesity (44), which will aggravate the condition. Also, the meta-analysis revealed a reduction in 15.65 min of sleep per day in PCOS women (**Figure 2C**), which indicated a shorter sleep duration. Sleep and body rhythm regulation play a critical role in normal metabolic health. Inadequate sleep or short sleep duration schedules can lead to metabolic disorders (45).

In the current study, we reported that sleep disturbance was related to higher BMI, SBP, DBP, WC, blood glucose, LDL-c, and lower HDL-c in PCOS women. A combination of BMI and WC may be a better parameter of obesity (46). In contrast to the finding of Winter et al. (47), data from the present analysis indicated that BMI was 6.86 kg/m^2^ and WC was 11.16 cm higher in participants with sleep disturbance, which reflected more obesity in PCOS women with sleep disturbance. One possible explanation is that sleep disturbance impairs signaling in the hypothalamic–pituitary–adrenal (HPA) axis, which can lead to changes in hormone levels. Dysregulation in the HPA axis is related to obesity (48). Another explanation is that obesity may affect the composition of the gut microbiota. According to the study conducted by Steegers-Theunissen and colleagues (49), PCOS is a brain disorder that should consider the role of the gut microbiota, which may influence central and hepatic clock gene expression and sleep duration in the host (50). Being overweight and obesity are also risk factors for OSA (51). All of these suggest that weight management interventions for PCOS may help improve sleep quality to a certain extent.

We found a significant relationship between sleep disturbance and impaired glucose regulation. Women with PCOS suffering from sleep disturbance had higher levels of fasting plasma glucose and 2-h glucose, both of which are known risk factors for cardiovascular disease. Changes in sleep architecture can lead to some shifts in glucose metabolism (14). When the levels of blood glucose are elevated, the body reduces metabolic stress through insulin resistance (52). Persistently high levels of serum glucose in patients with impaired glucose tolerance or diabetes will intensify insulin resistance and damage beta cells. In previous studies, insulin resistance was considered the cause of PCOS-induced sleep disturbance (10, 38), and disrupted sleep has also been associated with the improper treatment of insulin resistance. Insulin receptor substrates promote glucose uptake and intracellular protein synthesis *via* transmembrane glucose transporters, in which phosphorylation plays an important role (53). However, half of women have excessive serine phosphorylation of the insulin receptor (54, 55). This will aggravate insulin resistance in women with PCOS, amplify the need of beta cells to increase insulin secretion (56), and eventually lay a foundation for diabetes development. It was not surprising that PCOS with sleep disturbance would have worse lipid profiles and higher SBP and DBP. High-serum LDL-c and low-serum HDL-c were predictors of cardiovascular diseases (57). This suggests that poor sleep raises the hazard of cardiovascular diseases in PCOS. To summarize, sleep disturbance in women with PCOS is associated with a higher cardiovascular risk profile. Screening for cardiovascular disease in PCOS is critical.

The strength of this meta-analysis was that the comprehensive search strategy employed and the articles we included were from both developed and developing countries, and the participants came from different ethnicities, which could more fully reflect the sleep condition of PCOS from all over the world. The limitations of this study are as follows. Firstly, there was significant heterogeneity in our meta-analysis. The cultural differences in 18 trials from nine countries may have contributed to the heterogeneity. However, when we performed subgroup analysis, the heterogeneity of each group was decreased to 0%. Secondly, we did not report the specific sleep time per day of PCOS patients, and the specific PCOS average sleep duration still requires extensive clinical research. Thirdly, of the 18 included studies, 6 used subjective measures as their sleep outcomes, which could be considered inaccurate for measuring sleeping conditions. Lastly, most of the articles we included were cross-sectional studies, which cannot yet explain the causal relationship between PCOS and sleep disturbance, but only prove that there is a connection between the two issues. Further studies based on a large-sample, multicenter, randomized controlled trial design using polysomnography to measure sleep-related indicators are needed to help us draw a solid conclusion.

## Conclusion

In conclusion, the current study shows a high prevalence of sleep problems in women with PCOS and provides evidence of an association between cardiovascular risk factors and sleep disturbance among this population. Sleep assessment should be an integral part of the clinical guidelines for PCOS. Moreover, further research investigating the prevention of chronic metabolic disorders through the treatment of sleep disruption is also required.

## Data availability statement

The original contributions presented in the study are included in the article/supplementary material. Further inquiries can be directed to the corresponding authors.

## Author contributions

The study concept and design were framed by XC and CL. JZ and JY worked independently, scanned the titles and abstracts, and evaluated full text for eligible studies using Note-Express software. XC and CL resolved disagreements by discussion. JZ conducted the statistical data analysis and drafted the manuscript. XT and WL contributed to the discussion and revision. XC and CL contributed to the revision. All authors read and approved the final manuscript.

## Funding

This research was funded by the Natural Science Foundation of Fujian Province (No. 2020J011242), the Joint Research Project of Health and Education of Fujian Province (No. 2019-WJ-39), the Medical and Health Project of Xiamen Science & Technology Bureau (No. 3502Z20214ZD1001), and the Fujian Traditional Chinese Medicine Scientific Research Project (No. 2021zylc29).

## Acknowledgments

We are grateful to all the subjects for their participation.

## Conflict of interest

The authors declare that the research was conducted in the absence of any commercial or financial relationships that could be construed as a potential conflict of interest.

## Publisher’s note

All claims expressed in this article are solely those of the authors and do not necessarily represent those of their affiliated organizations, or those of the publisher, the editors and the reviewers. Any product that may be evaluated in this article, or claim that may be made by its manufacturer, is not guaranteed or endorsed by the publisher.
